# Association Between Inflammatory Bowel Disease and Pruritus

**DOI:** 10.1093/crocol/otaa012

**Published:** 2020-02-28

**Authors:** Shiho Iwamoto, Mitsutoshi Tominaga, Yayoi Kamata, Tomohiro Kawakami, Taro Osada, Kenji Takamori

**Affiliations:** 1 Juntendo Itch Research Center (JIRC), Institute for Environmental and Gender Specific Medicine, Juntendo University Graduate School of Medicine, Urayasu, Chiba, Japan; 2 Department of Gastroenterology, Juntendo University School of Medicine, Juntendo University Urayasu Hospital, Urayasu, Chiba, Japan; 3 Anti-aging Skin Research Laboratory, Juntendo University Graduate School of Medicine, Urayasu, Chiba, Japan; 4 Department of Dermatology, Juntendo University School of Medicine, Juntendo University Urayasu Hospital, Urayasu, Chiba, Japan

**Keywords:** dry skin, skin barrier dysfunction, inflammatory bowel disease, pruritus, itching, atopic dermatitis

## Abstract

**Background:**

Inflammatory bowel disease (IBD) is often complicated by extraintestinal manifestations. We frequently encounter IBD patients with pruritus; however, clinical evidence for the association of these conditions is lacking. Therefore, the present study investigated the incidence of pruritus in IBD patients.

**Methods:**

Seventy-one IBD outpatients, including 55 with ulcerative colitis (UC) and 16 with Crohn disease, and 39 healthy volunteers (HVs) were surveyed about their pruritus symptoms using a visual analogue scale (VAS). Disease activities in UC and Crohn disease patients were classified according to partial Mayo and IOIBD (International Organization for the Study of inflammatory Bowel Disease) scores, respectively. Skin barrier condition was examined by measuring transepidermal water loss and stratum corneum hydration. The distribution of intraepidermal nerve fibers in skin samples from 9 UC patients was examined immunohistochemically using an antiprotein gene product (PGP) 9.5 antibody.

**Results:**

Visual analogue scale scores were higher in IBD patients than in HV (*P* < 0.001). Active stage IBD patients had more severe pruritus VAS scores than those in the remission stage (*P* = 0.036). Transepidermal water loss was higher in IBD patients (*P* < 0.001) and active stage IBD patients (*P =* 0.004), while stratum corneum hydration was lower in IBD patients (*P =* 0.019) and active stage IBD patients than in HV (*P* = 0.019). A relationship was observed between the degree of pruritus and number of PGP9.5-immunoreactive intraepidermal nerve fibers in UC patients.

**Conclusions:**

Inflammatory bowel disease patients, particularly active stage patients, frequently exhibit symptoms of pruritus and dry skin. This result may have predictive and therapeutic implications for the treatment of IBD symptoms.

## INTRODUCTION

Inflammatory bowel disease (IBD) is often complicated by extraintestinal manifestations, including skin disorders, such as pyoderma gangrenosum, erythema nodosum, and erythema exudativum multiforme.^1^ Furthermore, although antitumor necrosis factor-α biologics are commonly used in the treatment of IBD and psoriasis, paradoxical adverse skin manifestations have been reported during biological treatments for IBD.^2^ These findings imply that some common immunoreactions are involved in IBD and its extraintestinal manifestations, particularly skin disorders. Recent studies have suggested several causes of IBD, such as genetic factors (eg, familial aggregation including twins and susceptibility genes), environmental factors (eg, industrialization and a diet high in fat and sugar), and disturbances in the enterobacterial flora inducing intestinal barrier loss.^3,4^ These causes, especially barrier dysfunction, are considered to play multiple important roles in the progression of IBD. Clinically, we frequently encounter IBD patients with pruritus and skin dryness; however, the most recent European Guidelines on Chronic Pruritus (2012) and other guidelines do not describe IBD-related itch.^5^ This is an important issue because pruritus worsens the quality of life of patients.^6^ Therefore, we investigated the relationship between IBD and dermatological complications, including pruritus.

## METHODS

### Patients and Control

Seventy-one IBD outpatients (males 39, females 32, mean age 40.87 ± 1.14 years, range 20–59 years), including 55 with ulcerative colitis (UC) (males 26, females 29), and 16 with Crohn disease (CD) (males 13, females 3) were examined. Controls comprised 39 healthy volunteers (HVs) (males 18, females 21, mean age 38.28 ± 1.36 years, range 25–58 years). (Table 1). Patients with dermatitis such as atopic dermatitis (AD) or chronic eczema were excluded.

**TABLE 1.** Characteristics of HV and IBD Patients

	HV	IBD	*P*
Total number of participants	39	71	
Age (y), mean ± SEM	38.28 ± 1.18	40.87 ± 1.14	ns
Breakdown			
Males/females	18/21 (46.1%/53.8%)	39/32 (55.0%/45.0%)	
UC/CD		55/16 (77.5%/5%)	
UC (M/F)		55 (M 26/F 29) (47.3%/52.7%)	
CD (M/F)		16 (M 13/F 3) (81.2%/18.8%)	
Therapeutic agent			
Mesalazine or salazosulfapyridine (yes/no)		59/12 (83.1%/16.9%)	
Prednisolone (yes/no)		12/59 (16.1%/83.1%)	
Biologics including infliximab, adalimumab, and golimumab (yes/no)		21/50 (29.3%/70.4%)	
Azathioprine (yes/no)		25/46 (35.2%/64.8%)	
IBD duration of disease since diagnosis, mean ± SEM		12.16 y ± 0.84	
Total Partial Mayo Index Score of UC (n = 55), mean ± SEM		1.93 ± 0.33	
IOIBD score of CD (n = 16), mean ± SEM		0.63 ± 0.22	

ns, nonsignificant.

### Evaluation of IBD, Pruritus, and Dry Skin

#### IBD

Disease activity was classified according to the noninvasive 9-point partial Mayo score for patients with UC and the International Organization for the Study of inflammatory Bowel Disease (IOIBD) score for those with CD.^7,8^ The partial Mayo Scoring Index requires the patient to score 2 items, stool frequency and rectal bleeding, on a scale from 0 to 3, and for the physician to similarly score their global assessment. UC severity was evaluated as the sum of the 3 scores (maximum score of 9 points). The IOIBD score included 10 items each scored as zero for absence and 1 for presence including (i) abdominal pain, (ii) bowel evacuation 6 times or more per day, (iii) anal lesion, (iv) fistula, (v) complications, (vi) abdominal tumor, (vii) body weight decrease, (viii) fever of 38°C or higher, (ix) abdominal tenderness, and (x) hemoglobin level of 10 g/dL or lower. The sum of the 10 items was used as the IOIBD score for patients with CD.

We classified UC patients using the total point score of the partial Mayo score: 0–1, remission; 2–4, mild disease; 5–6, moderate disease; and 7–9, severe disease. In CD patients, an IOIBD score of 0–1 was deemed “in remission,” otherwise the disease was deemed “active.” In the present study, since we were unable to perform endoscopy on every participant, disease activity was evaluated by clinical index only. The mean of the total partial Mayo score among 55 UC patients was 1.93 ± 0.33 (mean ± SEM), while that of the IOIBD index among 16 CD patients was 0.63 ± 0.22 (mean ± SEM).

#### Pruritus

We asked participants to self-assess their severity of pruritus. The worst case of pruritus anywhere on the whole body in the preceding 3 days was evaluated using a visual analogue scale (VAS). Participants were asked to rate their pruritus state using a 100-mm VAS graded from 0 (no pruritus) to 100 (worst imaginable pruritus). The severity of pruritus was classified as follows: no pruritus (VAS score = 0), mild 0 < VAS < 40 mm）, moderate (40 mm ≤ VAS < 70 mm), severe (70 mm ≤ VAS < 90 mm), and very severe (VAS ≥ 90 mm).^9^

#### Dry skin

The skin barrier condition of all participants was evaluated by noninvasive measures of transepidermal water loss (TEWL) and stratum corneum (SC) hydration of the radial aspect skin of the right forearm.^10^ Measurements were taken using VapoMeter and MoistureMeter SC Compact (Delfin Technologies Ltd., Kuopio, Finland), respectively. Those with presence of rashes, scratches, or other skin abnormalities in the measuring area or those that had applied any emollients in the measurement area were excluded. Since TEWL and SC hydration are often affected by temperature and humidity,^11^ we took measurements in an air-conditioned outpatient clinical room during the period from winter to early spring (between November and March) when it was relatively cool and dry in Japan. The average air temperature in the clinic (measurement room) was 26.5 ± 0.14°C (mean ± SEM) and relative humidity was 25.7% ± 1.45% (mean ± SEM).

### Eosinophil Count and Serum IgE Levels

We analyzed eosinophil counts and serum IgE to assess whether histamine was involved in induction of pruritus in IBD patients. Eosinophil counts were obtained using the ADVIA 2120i System (Siemens Healthineers AG, Munich, Germany) using peroxidase activity and nuclear density analysis cytograms. Blood samples were centrifuged at 3000 rpm at 20 to 25°C for 10 minutes. Data on serum IgE levels were analyzed using an electrochemiluminescence immunoassay with a Cobas 6000/e601 analyzer (F. Hoffmann-La Roche Ltd., Basel, Switzerland). The normal range of serum IgE was defined as 0–232 IU/mL.

### Immunohistochemistry

Three-millimeter punch biopsies were taken from the abdominal skin of 9 UC patients (males 5, females 4, mean age 42.67 ± SEM 2.74 years, range 30–53 years). They had no clinical skin abnormalities such as rashes or infection. The protocol for immunohistochemistry of epidermal nerve fibers was described previously.^12^ Briefly, to quantify the number of epidermal nerve fibers, 5–8 specimens per skin biopsy were stained with the antiproduct gene protein 9.5 (PGP9.5, 1:600; ENZO Life Science, Farmingdale, NY, USA). The numbers of PGP9.5-immunoreactive fibers penetrating into and lying within the epidermis are shown as the means of 5–8 slices.

### Statistical Analysis

Pruritus VAS scores, TEWL, SC hydration, IBD disease activity, and other results were analyzed using GraphPad Prism7 (GraphPad Software, La Jolla, CA, USA). Between group differences were analyzed using 2-tailed Mann–Whitney test with *P* < 0.05 deemed significant, and Spearman correlation test was used to assess correlations among data with coefficients (*r*) 0.2 to 0.4, >0.4 to 0.7, and >0.7 deemed weak, moderate, and strong, respectively.

### Ethical Considerations

The present study was conducted according to the principles of the Declaration of Helsinki and was approved by the Medical Ethical Committee of Juntendo University Urayasu Hospital. All study participants provided written informed consent.

## RESULTS

**FIGURE 1.**
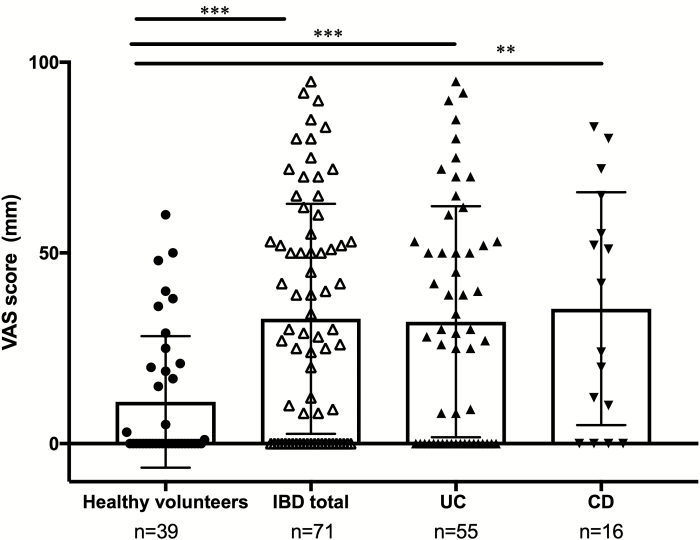
Visual analogue scale scores for the degree of pruritus. Visual analogue scale scores in HV (n = 39) and IBD patients (n = 71), including UC (n = 55) and CD (n = 16). *P* values were obtained by 2-tailed Mann–Whitney test, 95% confidence level, significance *P* < 0.05. *P* value style: 0.002(**), and <0.001(***).

The mean VAS score was significantly higher in IBD (mean VAS = 32.74 ± SEM 3.58, *P* < 0.001), UC (mean VAS = 31.96 ± SEM 4.08 *P* < 0.001), and CD patients (mean VAS = 35.38 ± SEM 7.63, *P* = 0.002) than in HV (mean VAS = 10.95 ± SEM 2.76). Notably, 41.0% of HV with no dermatological disorders reported pruritus. A total of 67.6% of IBD, 65.5% of UC, and 75.0% of CD patients also reported pruritus (Fig. 1 and Supplementary Table S1(i)).

**FIGURE 2.**
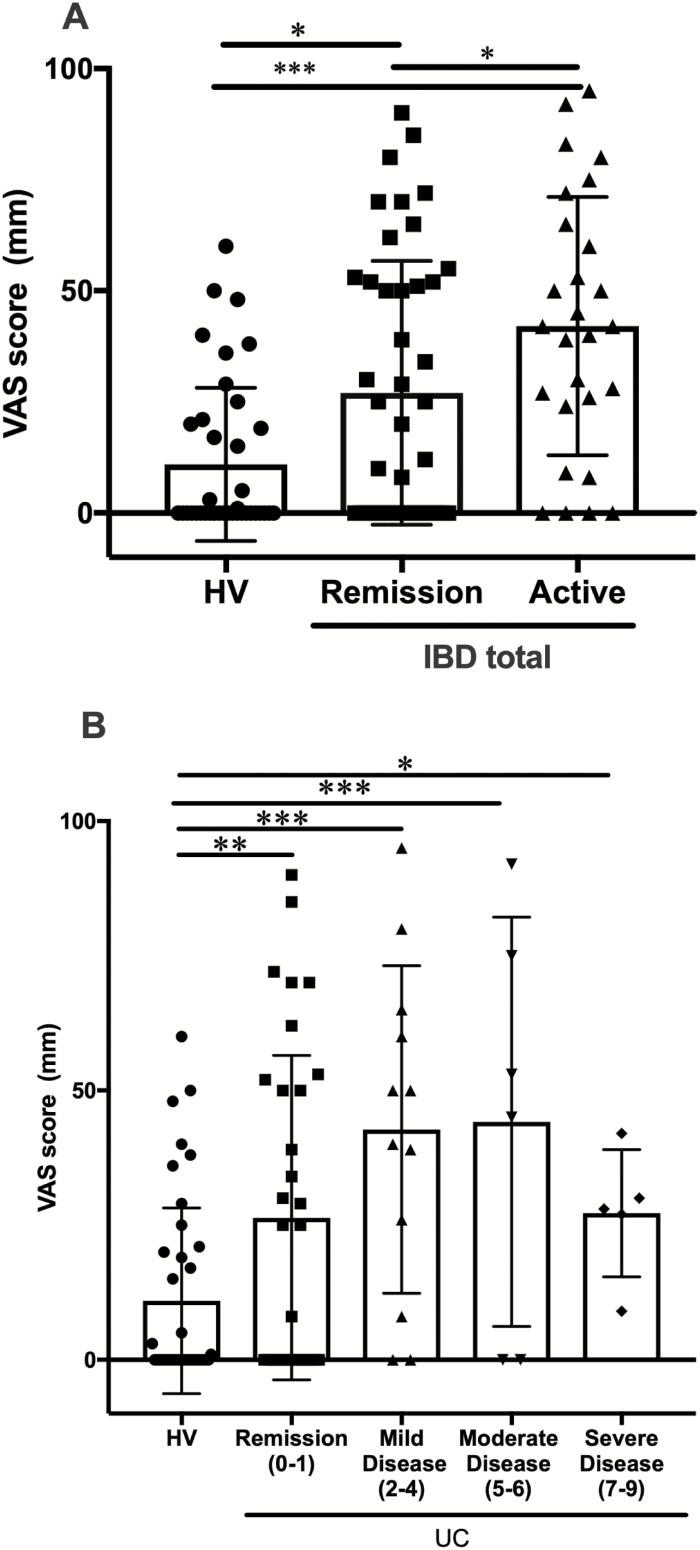
Relationship between disease activity and pruritus in IBD (A) and UC (B). A, The relationship between disease activity and pruritus in IBD patients. Remission IBD patients (n = 44), active IBD patients (n = 27), and HV (n = 39). B, The relationship between disease activity and pruritus in UC patients. Activity levels in patients with UC (n = 55) were classified by the total partial Mayo score as follows: remission (n = 32), mild (n = 12), moderate (n = 6), and severe disease (n = 5). Visual analogue scale scores of all these groups were significantly higher than those of HV. *P* value style: 0.033(*), 0.002(**), and <0.001(***).

We then investigated the relationship between disease activity and pruritus in IBD patients (Fig. 2). IBD patients in active (mean VAS = 42.05 ± SEM 5.59, *P* < 0.001) and remission phases (mean VAS = 27.02 ± SEM 4.47, *P =* 0.018) showed more severe pruritus than HV (Fig. 2A). In addition, active phase IBD patients showed more severe pruritus than remission phase patients (*P =* 0.036) (Fig. 2A). In 55 UC patients, a relationship was observed between VAS scores and disease severity using the total partial Mayo score. Remission (mean VAS = 26.38 ± SEM 5.47, n = 32, *P* = 0.009), mild (mean VAS = 42.75 ± SEM 8.72, n = 12, *P* < 0.001), moderate (mean VAS = 44.17 ± SEM 15.51, n = 6, *P* < 0.001), and severe disease patients (mean VAS = 27.20 ± SEM 5.27, n = 5, *P* = 0.048) showed significantly higher VAS scores than HV, with the moderate disease group showing the highest mean VAS score (Fig. 2B).

**FIGURE 3.**
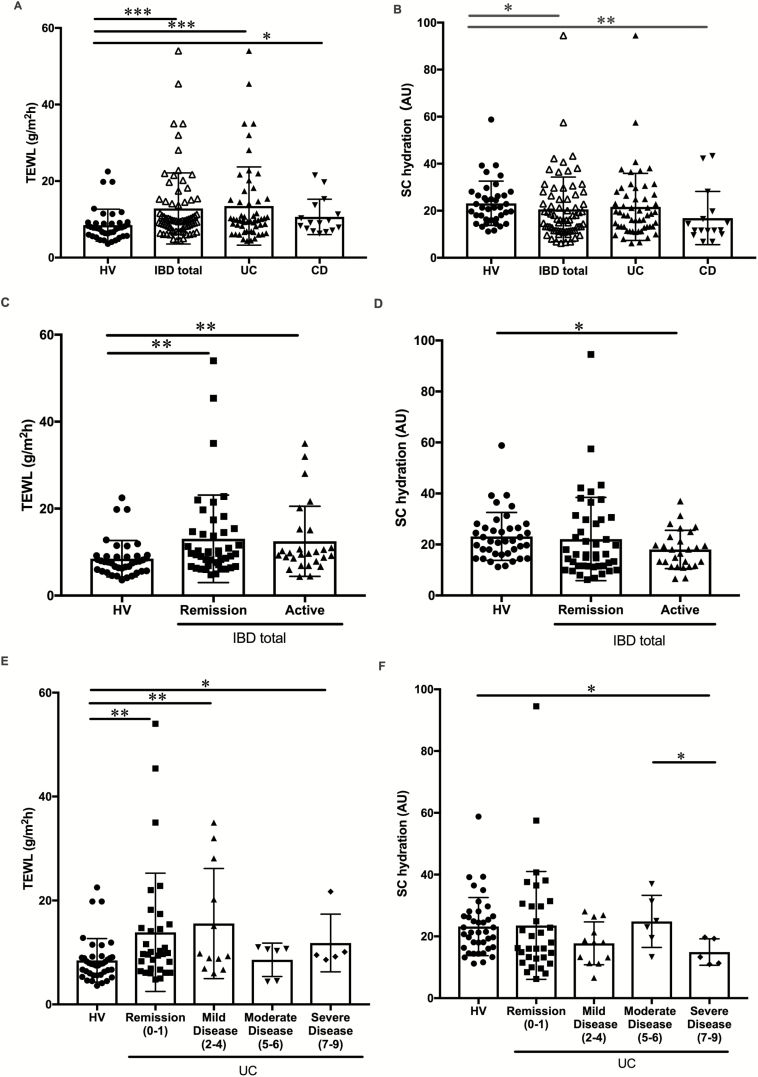
Transepidermal water loss (A, C, E) and SC hydration (B, D, F). A, TEWL in patients with IBD (n = 71), UC (n = 55), and CD (n = 16) and in HV (n = 39). B, SC hydration in patients with IBD, UC, and CD and in HV. C, TEWL in IBD patients in remission (n = 44) and the active phase (n = 27) and in HV (n = 39). D, SC hydration in IBD patients in remission and the active phase and in HV. E, F, TEWL (E) and SC hydration (F) in the UC group classified by the total partial Mayo score; remission (n = 32), mild (n = 12), moderate (n = 6), and severe disease (n = 5). *P* value style: 0.033(*), 0.002(**), and <0.001(***).

Skin barrier condition was examined on the forearm by measuring TEWL and SC hydration. TEWL was significantly higher in IBD (mean TEWL = 12.84 ± SEM 1.10, *P* < 0.001), UC (mean TEWL = 13.48 ± SEM 1.38, *P* < 0.001), and CD patients (mean TEWL = 10.65 ± SEM 1.16, *P* = 0.04) than in HV (mean TEWL = 8.48 ± SEM 0.67) (Fig. 3A). SC hydration was significantly less in IBD (mean SC hydration = 20.57 ± SEM 1.63, *P* = 0.019) and CD patients (mean SC hydration = 16.89 ± SEM 2.83, *P =* 0.013) than in HV (mean SC hydration = 23.18 ± SEM 1.51) No significant difference was observed in SC hydration between UC (mean SC hydration = 21.64 ± SEM 1.92, *P* = 0.98) and HV (Fig. 3B). IBD patients showed a higher TEWL than HV in both the active (mean TEWL = 12.49 ± SEM 1.55, *P =* 0.004) and remission (mean TEWL = 13.06 ± SEM 1.55, *P =* 0.002) phases (Fig. 3C). IBD patients also showed less SC hydration than HV in the active phase (mean SC hydration = 18.03 ± SEM 1.44, *P* = 0.019). However, there was no significant difference in SC hydration between patients in the remission phase (mean SC hydration = 22.13 ± SEM 2.46, *P* = 0.73) and HV (Fig. 3D). Moreover, TEWL and SC hydration in the active and remission phases were not significantly different (Figs. 3C, D). We also classified TEWL and SC hydration by total partial Mayo scores in 55 UC patients (Figs. 3E, F). Remission (*P* = 0.004), mild (*P* = 0.009), and severe disease groups (*P* = 0.020) showed significantly higher TEWL than HV. Only severe disease patients showed less SC hydration than HV (*P* = 0.020). No significant difference was observed in SC hydration between the other activity category and HV (Fig. 3F). However, a significant difference was noted between SC hydration in the moderate and severe disease groups (*P* = 0.030) (Fig. 3F) (see Supplementary Table S1(i), (iii), (iv) in detail).

No significant difference was observed in eosinophil counts between the HV and IBD groups (Supplementary Table S2(i)). No significant difference was observed in serum IgE levels between the HV and IBD groups; however, a weak negative correlation was noted between serum IgE levels and SC hydration in HV only (Supplementary Table S2(ii)). Furthermore, no significant difference was observed in VAS scores, TEWL, or SC hydration between the sexes (Supplementary Table S1(ii)). Eighty-four (83.0%) IBD patients in the present study were administered mesalazine or salazosulfapyridine, 12 (16.9%) prednisolone, 21 (29.5%) biologic agents, and 25 (35.2%) Azathioprine. A significant difference (*P* = 0.034) was observed in SC hydration only between patients treated with or without Azathioprine (Table 1 and Supplementary Table S1(v)).

The medication of IBD is shown in Table 1. None of the IBD patients had any specific extraintestinal skin manifestations. The mean duration of disease following diagnosis of IBD was 12.16 ± 0.84 (mean ± SEM) years (Table 1).

**FIGURE 4.**
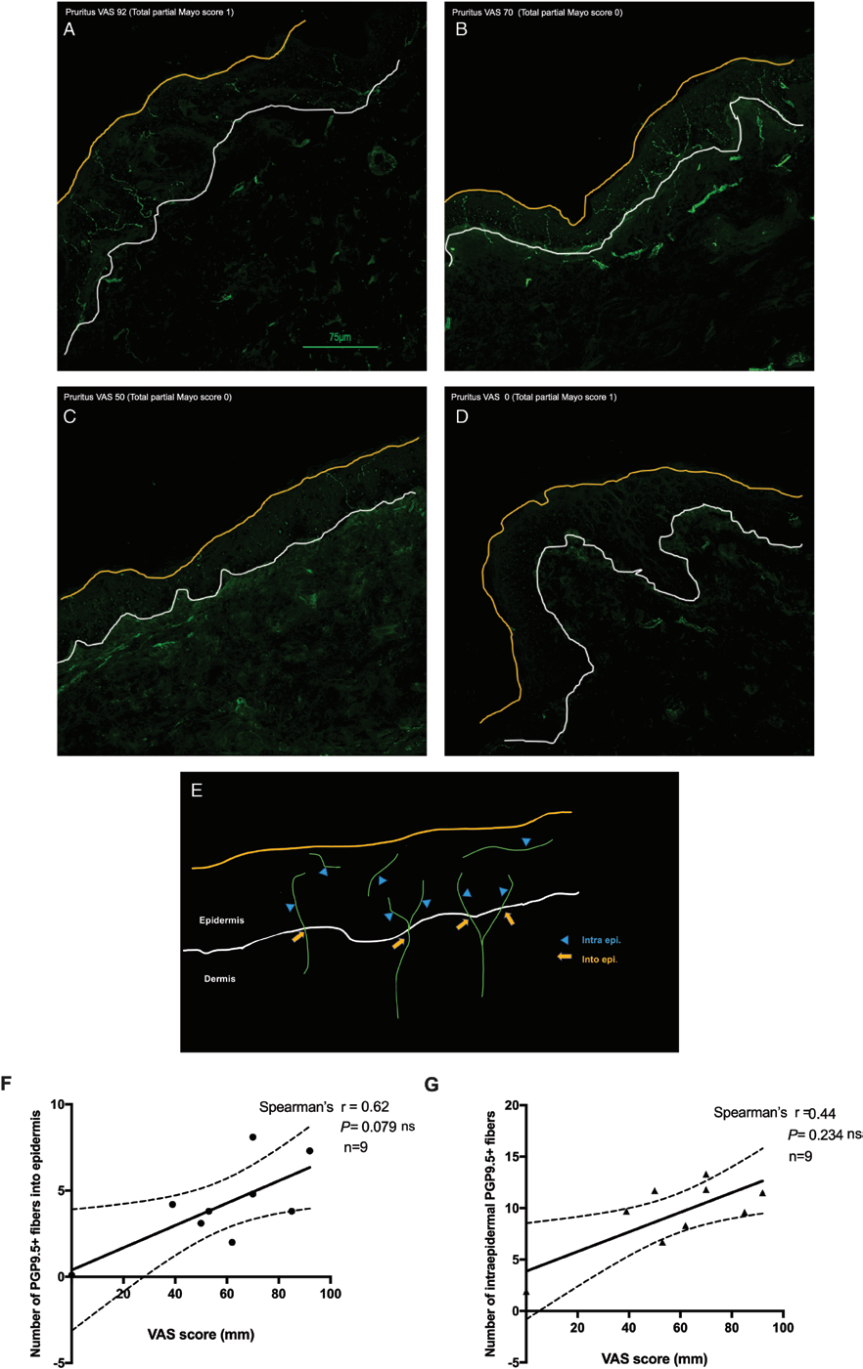
Intraepidermal nerve fibers in UC. A–D, Representative immunohistochemical staining of skin samples from UC patients with an antibody to PGP9.5. PGP9.5-immunoreactive (PGP9.5^+^) fibers (green) were occasionally present in the epidermis and dermis. Higher densities of epidermal nerve fibers were observed in UC patients with pruritus. Scale bar = 75 µm. E, A schematic of the number of intraepidermal PGP9.5^+^ fibers (blue arrowheads) and PGP9.5^+^ fibers that penetrated the epidermis (yellow arrows). F–G, A positive association was observed between VAS scores and the number of intraepidermal PGP9.5^+^ fibers (F) or number of PGP9.5^+^ fibers that penetrated the epidermis.

The distribution of intraepidermal nerve fibers (IENFs) in the skin of 9 UC patients with pruritus was examined immunohistochemically using the anti-PGP9.5 antibody (Fig. 4). PGP9.5-immunoreactive fibers in the epidermis and intraepidermal area exhibited higher densities in UC patients with pruritus (Figs. 4A–C) than in those without pruritus (Fig. 4D). The VAS score was associated with the number of PGP9.5-immunoreactive fibers in the epidermis (Fig. 4F) and intraepidermal area (Fig. 4G). No relationships were observed among the number of IENF, TEWL, SC hydration, or total partial Mayo score (Supplementary Table S3).

## DISCUSSION

Few studies have attempted to evaluate pruritus in IBD patients. The present study showed that the mean VAS score was significantly higher in IBD patients than in HV, and 68% of IBD patients had mild to very severe pruritus (Fig. 1). Furthermore, TEWL increased, while SC hydration decreased in IBD patients (Figs. 3A, B). This result suggests that IBD patients had dry skin due to barrier dysfunction. Patients in the present study were recruited from an outpatient clinic, and their IBD clinical conditions were generally mild (see Table 1, mean of the total partial Mayo score and IOIBD score). We speculate that their dry skin condition was not related to dehydration due to frequent diarrhea. Dry skin induces pruritus. Therefore, these results suggest that most IBD patients manifest dry skin with pruritus.^13,14^ Pruritus worsens the quality of life of patients in addition to the discomfort associated with IBD,^15,16^ therefore pruritus needs to be considered in the management of IBD patients.

Previous studies demonstrated that dry skin induces itching, which is resistant to conventional treatments, such as histamine H_1_ receptor antagonists.^17^ Furthermore, increased sensory nerve density in the epidermis is partly involved in itching hypersensitivity, such as xerosis and AD.^17^ In the present study, we found increased IENF in UC patients, which was similar to dry skin features in AD. In a recent study, although the patient not noted as having a typical dermatological complication of IBD the study^18^ reported a bidirectional association between AD and IBD. This finding raises the possibility that there is a close relationship between skin barrier and intestinal conditions.

Moreover, our immunohistochemical findings showed a relationship between the degree of pruritus and number of IENF in UC patients (Fig. 4). In contrast, no significant differences were observed in serum IgE levels or the eosinophil count between HV and IBD patients (Supplementary Table S2(i), (ii)). These results suggest that many IBD patients frequently have dry skin and pruritus without marked dermatological manifestations and that itch in IBD patients is histamine independent.

The present results revealed that the disease activities of IBD correlated with the degree of pruritus, TEWL, and SC hydration (Figs. 2A and 3C, D). Moreover, the degree of pruritus and TEWL were significantly higher in IBD patients than in HV, even in the remission phase (Figs. 2A and 3C). These results imply that IBD patients have a dysfunctional skin barrier, and their disease activity reflects the exacerbation of dry skin and pruritus.

We found the highest mean VAS score was in the moderate disease UC group rather than the severe disease group (Fig. 2A). No correlation was observed between VAS score and total partial Mayo score in all UC patients (Spearman *r* = 0.21, *P* = 0.119, n = 55, Supplementary Fig. S1). Moreover, statistical analyses revealed a negative relationship between total partial Mayo scores and the number of PGP9.5-IENF in UC patients (Spearman *r* = −0.358, *P* = 0.36, n = 9, Supplementary Table S3). Therefore, although the number of skin biopsy samples examined was small, UC-associated pruritus may be an indicator of the worsening of symptoms in patients. A recent study on CD reported that extraintestinal manifestations, including skin disorders, occurred at a lower frequency in elderly onset than in pediatric-onset patients.^19^ Moreover, increased TEWL in IBD patients indicates skin barrier dysfunction, and an allergic reaction due to percutaneous sensitization for certain allergens may occur.^20,21^ If IBD patients have skin barrier dysfunction, it may be associated with an immunoreaction due to percutaneous sensitization in addition to disruption of intestinal mucosal barrier and disturbance of intestinal flora. Therefore, our findings imply that IBD patients may present with dysfunction of multiple epithelial barriers.

One of the limitations of the present study was that we were unable to perform colonoscopy on patients at the same time as assessing their skin condition. Thus, we did not investigate the relationship between mucosal and skin barrier dysfunctions. In addition, the serum analysis was limited by national health insurance coverage. In this study, the combination of IBD patient’s medication was diverse even in remission, therefore the number in each group was too small to consider the effect of medications. Moreover, we were unable to perform skin biopsy on HV with pruritus to compare the distribution of IENF in their skin with that of IBD patients.

## CONCLUSIONS

The results of the present study suggest that IBD patients have dry skin features and frequently develop pruritus. These symptoms need to be recognized as clinical complications in IBD patients.

## Supplementary Material

otaa012_suppl_Supplementary_Legends

otaa012_suppl_Supplementary_Table_1

otaa012_suppl_Supplementary_Table_2

otaa012_suppl_Supplementary_Table_3

## Notes

Author Contribution: S.I. performed the experiments and wrote the manuscript. S.I., M.T., Y.K., and K.T. designed the research study. All authors collaborated on the research results.

Funding: This work was supported by a grant from the Strategic Research Foundation Grant-aided Project for Private Universities from the Ministry of Education, Culture, Sports, Science and Technology (MEXT) (S1311011).
