# Supplementary material for: Association Between Inflammatory Bowel Disease and Pruritus
Source: Crohns Colitis 360. 2020 Feb 28;2(1):otaa012. doi: 10.1093/crocol/otaa012 (PMC9802076; doi:10.1093/crocol/otaa012)
Supplement: otaa012_suppl_Supplementary_Legends [file otaa012_suppl_supplementary_legends.docx]

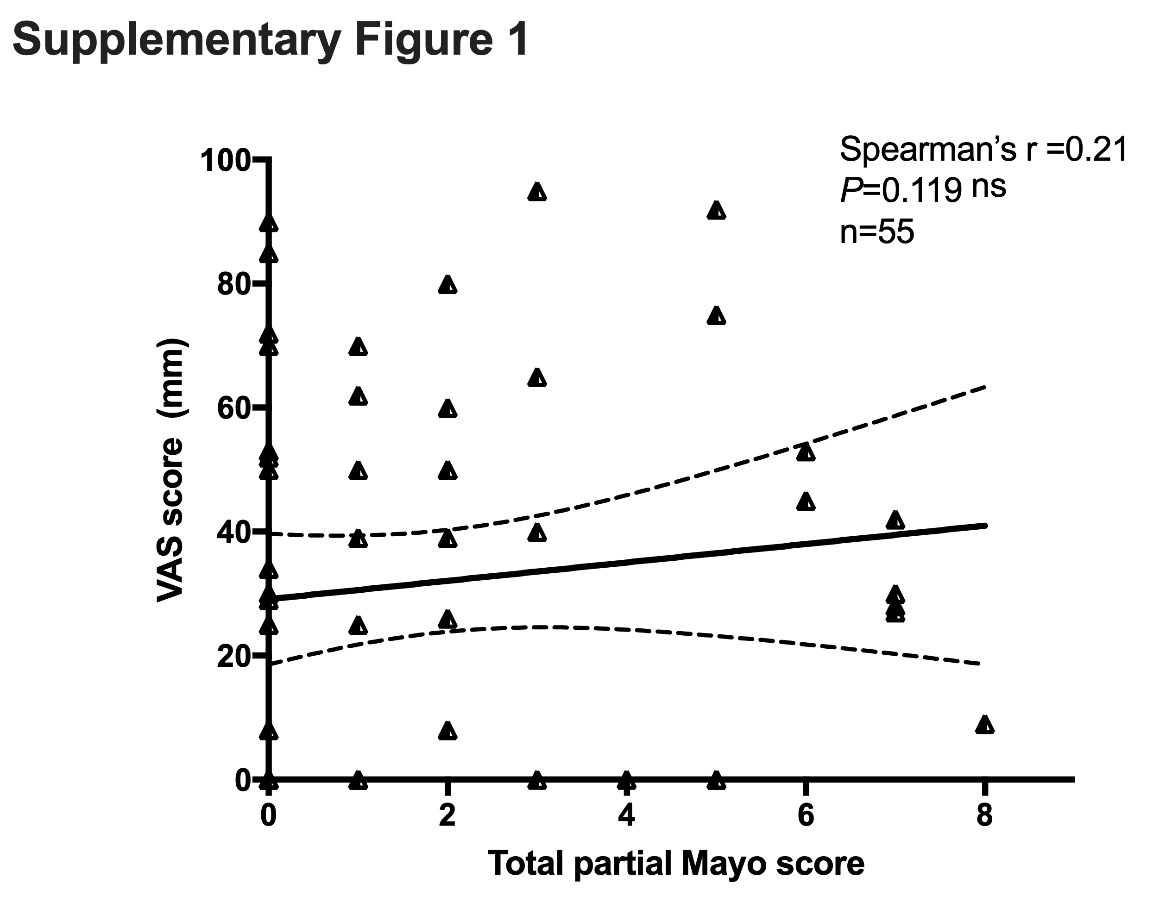


**Supplementary Figure 1**

Correlation analysis between the degree of pruritus and total partial Mayo Scores.

No correlation was discovered (Spearman’s r=0.21, *P=*0.119 ns, n=55).
