# Supplementary material for: Association Between Inflammatory Bowel Disease and Pruritus
Source: Crohns Colitis 360. 2020 Feb 28;2(1):otaa012. doi: 10.1093/crocol/otaa012 (PMC9802076; doi:10.1093/crocol/otaa012)
Supplement: otaa012_suppl_Supplementary_Table_1 [file otaa012_suppl_supplementary_table_1.pdf]

Supplementary Table 1. Statistical analyses of VAS score, TEWL value and SC hydration by sex, disease activities and medication.

		VAS score (mm)							TEWL value (g/m^2h)							SCH (AU)						
(i)	Number	Mean	Median	Std. Deviation	Std. Error of Mean	Lower 95% CI of mean	Upper 95% CI of mean	MW -TEST	Mean	Median	Std. Deviation	Std. Error of Mean	Lower 95% CI of mean	Upper 95% CI of mean	MW-TEST	Mean	Median	Std. Deviation	Std. Error of Mean	Lower 95% CI of mean	Upper 95% CI of mean	MW-TEST
Healthy volunteers (HV)	39	10.95	0.0	17.23	2.76	5.36	16.54		8.48	7.8	4.19	0.67	7.12	9.840		23.18	21.3	9.40	1.51	20.13	26.22	
IBD total	71	32.75	29.0	30.15	3.58	25.60	39.87	*P*<0.001(vs. HV)***	12.84	9.7	9.30	1.10	10.64	15.04	*P*<0.001(vs. HV)***	20.57	16.2	13.73	1.63	17.32	23.82	*P*=0.019(vs. HV)*
UC	55	31.96	29.0	30.28	4.08	23.78	40.15	*P*<0.001(vs. HV)***	13.48	9.8	10.22	1.38	10.72	16.24	*P*<0.001(vs. HV)***	21.64	18.1	14.27	1.92	17.79	25.50	*P*=0.98(vs. HV) ns
CD	16	35.38	33.0	35.38	7.64	19.10	51.65	*P*=0.002(vs. HV)**	10.65	9.1	4.62	1.16	8.186	13.11	*P*=0.040(vs. HV)*	16.89	11.7	11.33	2.83	10.86	22.93	*P*=0.013vs. HV)**
(ii)																						
HV Males	18	5.00	0.0	12.66	2.99	-1.30	11.30	*P*=0.64ns (vs females)	9.89	8.1	5.37	1.27	7.22	12.57	*P*=0.16 ns (vs female)	21.61	21.3	6.78	1.60	18.24	24.98	*P*=0.52ns (vs female)
HV Females	21	16.05	5.0	19.21	4.19	7.31	24.79		7.27	6.7	2.34	0.51	6.21	8.34		24.52	22.9	11.17	2.44	19.43	29.60	
IBD Males	39	31.10	26.0	30.00	4.83	21.38	23.65	*P*=0.72 ns(vs females)	14.44	10.1	11.23	1.80	10.8	18.08	*P*=0.34 ns (vs female)	19.80	16.1	15.36	2.46	14.82	24.78	*P*=0.24ns (vs female)
IBD Females	36	24.72	34.5	30.70	5.43	23.65	45.79		10.89	9.6	5.786	1.02	8.805	12.98		21.52	18.1	11.62	2.05	17.33	25.70	
(iii)																						
IBD Active	27	42.04	42.0	29.08	5.60	30.53	53.54	*P*< 0.001(vs. HV)***	12.49	9.7	8.05	1.55	9.31	15.67	*P*=0.004(vs. HV)**	18.03	18.1	7.502	1.44	15.06	21.00	*P*=0.019(vs. HV)*
IBD Remission	44	27.02	16.0	29.68	4.47	18.00	36.04	*P*=0.018(vs. HV)*, *P*=0.036(vs. Active)*	13.06	9.7	10.08	1.52	9.99	16.12	*P*=0.002(vs. HV)**, *P*=0.95(vs. Active) ns	22.13	16.1	16.32	2.46	17.17	27.09	*P*=0.075(vs. HV) ns *P*=0.73 (vs. Active) ns
(iv)																						
UC Remission (Total partial Mayo score 0-1)	32	26.38	16.5	30.13	5.326	15.51	37.24	*P*=0.009(vs. HV)**	13.87	9.8	11.38	2.011	9.77	17.97	*P*=0.004(vs. HV)**	23.54	17.1	17.46	3.09	17.25	29.84	*P*=0.26(vs. HV) ns
MIld disease (score 2-4)	12	42.75	45.0	30.42	8.782	23.42	62.08	*P*<0.001(vs. HV)***	15.57	9.6	10.59	3.06	8.84	22.30	*P*=0.009(vs. HV)**	17.77	18.1	6.93	2.00	13.36	22.17	*P*=0.08vs. HV) ns
Moderate disease (score 5-6)	6	44.17	49.0	38.00	15.51	4.29	84.04	*P*<0.001(vs. HV)***	8.583	10.5	3.21	1.31	5.22	11.95	*P*=0.61(vs. HV) ns	24.85	24.0	8.42	3.44	16.01	33.69	*P*=0.52(vs. HV) ns
Severe disease (score 7-9)	5	27.20	28.0	11.82	5.29	12.52	41.88	*P*=0.048(vs. HV)*	11.82	9.5	5.55	2.482	4.93	18.71	*P*=0.02(vs. HV)*	14.92	13.3	4.28	1.91	9.611	20.23	*P*=0. 02(vs. HV)*, *P* =0.03*(vs moderate)
(v)																						
With Mesalazine/ Salazosulfapyridine	59	31.34	27.0	31.13	4.05	23.23	39.45		13.08	9.7	9.816	1.28	10.53	15.64		20.76	16.5	14.44	1.88	17.00	24.52	
Without Mesalazine/ Salazosulfapyridine	12	39.58	46.0	24.79	7.16	23.83	55.34	*P*=0.32 ns(vs with 5ASA/SASP)	11.64	9.6	6.374	1.84	7.59	15.69	*P*=0.89 ns(vs with 5ASA/SASP)	19.66	15.6	9.991	2.88	13.31	26.01	*P*=0.92 ns(vs with 5ASA/SASP)
With Prednisolone	12	25.42	25.0	25.79	7.45	9.03	41.81		9.717	7.4	6.26	1.81	5.74	13.69		17.53	16.5	7.083	2.05	13.02	22.03	
Without Prednisolone	59	34.22	30.0	30.94	4.03	26.16	42.28	*P*=0.41 ns(vs with Steroids)	13.48	9.9	9.72	1.27	10.94	16.01	*P*=0.07 ns(vs with Steroid)	21.19	16.2	14.69	1.91	17.36	25.02	*P*=0.76 ns(vs with Steroid)
With Biologics	21	37.57	42.0	29.2	6.37	24.28	50.86		13.90	10.3	9.522	2.08	9.57	18.24		17.65	14.4	9.345	2.04	13.40	21.91	
Witout Biologics	50	30.70	26.0	30.6	4.33	22.00	39.40	*P*=0.34ns(vs with Biologics)	12.39	9.7	9.268	1.31	9.76	15.03	*P*=0.42 ns(vs with Biologics)	21.8	18.1	15.12	2.14	17.5	26.09	*P*=0.33 ns(vs with Biologics)
With Azathioprine	25	32.04	24.0	33.74	6.747	18.11	45.97		14.11	9.8	10.86	2.172	9.63	18.59		18.47	13.2	17.31	3.46	11.32	25.61	
Without Azathioprine	46	33.11	30.0	28.4	4.19	24.67	41.54	*P*=0.78 ns(vs with AZP)	12.15	9.7	8.38	1.24	9.66	14.64	*P*=0.38 ns(vs with AZP)	21.72	19.4	11.38	1.68	18.33	25.10	*P*=0.034*(vs with AZP)
