# Supplementary material for: Association Between Inflammatory Bowel Disease and Pruritus
Source: Crohns Colitis 360. 2020 Feb 28;2(1):otaa012. doi: 10.1093/crocol/otaa012 (PMC9802076; doi:10.1093/crocol/otaa012)
Supplement: otaa012_suppl_Supplementary_Table_2 [file otaa012_suppl_supplementary_table_2.pdf]

Supplementary Table 2. Comparison of eosinophils and IgE in HV vs IBD.

(i)

Eoshinophils	Number	Eosinophils count Mean ± SEM	Median	Std. Deviation	Range	Eoshinophils mean HV vs IBD M-W test	Correlation of VAS score Spearman' s r	P(two-tailed)	Correlation of TEWL Spearman's r	P(two-tailed)	Correlation of SCH Spearman's r	P(two-tailed)
Healthy volunteers	39	178.9 ± 31.49	140.4	196.6	32.9 to 974.9	*P* =0.10 ns	-0.005	0.98 ns	0.077	0.64 ns	-0.065	0.69 ns
IBD total	71	208.0± 18.29	163.0	154.1	15.0to 713.8		-0.1803	0.13ns	0.043	0.72 ns	0.0584	0.63ns

(ii)

IgE	Number	Serum IgE Mean ± SEM	Median	Std. Deviation	Range	Serum IgE HV vs IBD M-W test	Correlation of VAS score Spearman's r	P(two-tailed)	Correlation of TEWL Spearman' s r	P(two-tailed)	Correlation of SCH Spearman's r	P(two-tailed)
Healthy volunteers	39	190.5 ± 54.50	62.1	340.3	3.2 to 1990	*P* =0.62 ns	-0.013	0.93 ns	0.105	0.53 ns	-0.331	0.039*
IBD total	43	167.0± 42.76	71.9	280.4	1.2 to1346		-0.1241	0.48ns	-0.241	0.12 ns	-0.029	0.85 ns
