# Supplementary material for: Association Between Inflammatory Bowel Disease and Pruritus
Source: Crohns Colitis 360. 2020 Feb 28;2(1):otaa012. doi: 10.1093/crocol/otaa012 (PMC9802076; doi:10.1093/crocol/otaa012)
Supplement: otaa012_suppl_Supplementary_Table_3 [file otaa012_suppl_supplementary_table_3.pdf]

Supplementary Table 3. Comparison of PGP 9.5-positive nerve fibers with VAS score, TEWL value and SC hydration.

	Sample numbers	Nerve fibers count Mean ± SEM	Std. Deviation	Median	Lower 95% CI of mean	Upper 95% CI of mean	Correlation of VAS score Spearman's r	P(two-tailed)	Correlation of TEWL Spearman's r	P(two-tailed)	Correlation of SCH Spearman's r	P(two-tailed)	Correlation of the partial Mayo score	P(two-tailed)
PGP 9.5-positive nerve fibers into epidermis	9	4.13 ± 0.82	2.46	3.8	2.24	6.02	0.622	0.08 ns	0.109	0.78 ns	-0.067	0.87 ns	-0.097	0.79 ns
Intraepidemal PGP 9.5-positive nerve fibers	9	9.39 ± 1.15	3.46	9.7	6.73	12.05	0.444	0.23 ns	0.500	0.17ns	-0.333	0.39 ns	-0.358	0.36 ns
